# Robot-assisted Repair of Rectovesical Fistula after Radical Prostatectomy using the Hugo™ RAS System

**DOI:** 10.1590/S1677-5538.IBJU.2025.0405

**Published:** 2025-09-15

**Authors:** Lara Herrero López, Andrea Noya Mourullo, Sara Tamburini, Edoardo Beatrici, Nicola Frego, Simone Morra, Florencio Manuel Marin Martinez, Geert De Naeyer, Ruben De Groote, Edward Lambert, Frederiek D’Hondt, Alexandre Mottrie

**Affiliations:** 1 AZORG Hospital Department of Urology Merelbeke-Melle Belgium Department of Urology, AZORG Hospital, Merelbeke-Melle, Belgium; 2 ORSI Academy Merelbeke-Melle Belgium ORSI Academy, Merelbeke-Melle, Belgium; 3 Department of Urology, CHU Brugmann Brussels BE Belgium Department of Urology, CHU Brugmann, Brussels, BE; 4 UROINTEC Urología Innovación Tecnología Department of Urology España Department of Urology, UROINTEC Urología Innovación Tecnología, Islas Canarias, España; 5 Reproductive Sciences and Odontostomatology University of Naples Federico II Department of Neuorsciences Naples Italy Department of Neuorsciences, Reproductive Sciences and Odontostomatology University of Naples Federico II, Naples, Italy; 6 Hospital General Universitario Santa Lucía Department of Urology Murcia Spain Department of Urology, Hospital General Universitario Santa Lucía, Cartagena, Murcia, Spain

## Abstract

**Introduction:**

Rectovesical fistula (RVF) is a rare complication after robot-assisted radical prostatectomy (RARP) (1), often requiring complex surgery (2). Robotic systems provide dexterity and visualization for deep pelvic procedures (3, 4). We report the first RVF repair using the Hugo™ RAS System.

**Materials and Methods:**

A 76-year-old male developed fecaluria one week after catheter removal following RARP. MRI revealed a 1.3 cm fistulous tract between the bladder and rectum. Initial management included transurethral and suprapubic catheters, plus a loop colostomy. Robotic repair was performed five months later. Trocar placement, adapted to the stoma, included four robotic and two assistant ports. Posterior bladder wall dissection allowed removal of two joined catheters. The posterior bladder wall, urethrovesical anastomosis dehiscence, and a 1 cm anterior rectal defect were repaired. Fibrotic tissue and residual clip were removed. A peritoneal flap was interposed between the bladder and rectum, and a new bladder neck and vesicourethral anastomosis were created using barbed sutures. Intraoperative testing confirmed integrity, and a bladder catheter was placed.

**Results:**

The postoperative course was uneventful, with patient discharge on day 4. The bladder catheter was removed after 3 weeks. At the 2-month follow-up, urinary function was normal with good continence. Ultrasound confirmed good bladder filling and no post-void residual. Cystoscopy showed a well-healed urethrovesical anastomosis without fistula. Colostomy reversal is pending.

**Conclusions:**

This case demonstrates the feasibility and effectiveness of the Hugo™ RAS System for RVF repair post-RARP. Robotic surgery can manage complex defects with favorable outcomes (5). Robotic platforms may expand telesurgery, allowing patients to undergo procedures locally with expert surgeons operating remotely (6).

**Available at:**VIDEO

## Data Availability

Uninformed
